# Treatment and outcomes of primary mediastinal B cell lymphoma: a three-decade monocentric experience with 151 patients

**DOI:** 10.1007/s00277-020-04364-0

**Published:** 2020-12-10

**Authors:** Beatrice Casadei, Lisa Argnani, Alice Morigi, Ginevra Lolli, Alessandro Broccoli, Cinzia Pellegrini, Laura Nanni, Vittorio Stefoni, Paolo Elia Coppola, Matteo Carella, Michele Cavo, Pier Luigi Zinzani

**Affiliations:** 1 IRCCS - Azienda Ospedaliero-Universitaria di Bologna, Bologna, Italy; 2 Institute of Hematology “L. e A. Seràgnoli”, University of Bologna, Bologna, Italy

**Keywords:** Primary mediastinal B cell lymphoma, MACOP-B, Rituximab, Radiotherapy, Checkpoint inhibitor

## Abstract

Primary mediastinal B cell lymphoma is a rare entity and often should be promptly treated as a hematological emergency: The initial treatment decision is crucial for the management of this disease. An observational retrospective study was conducted with the aim to improve information on treatment and outcomes of primary mediastinal B cell lymphoma in real practice. After 12 cycles of MACOP-B regimen (methotrexate, doxorubicin, cyclophosphamide, vincristine, bleomycin, and prednisone) with or without rituximab, 120 patients out of 151 (79.5%) achieved a complete response and 12 (7.9%) a partial response leading to a global response of 87.4%. The 21-year overall survival is 82.6%; progression-free and disease-free survivals are 69.3% and 86.4%, respectively. Regarding the role of radiotherapy (RT), patients with a negative PET scan after MACOP-B did not undergo RT: One out of these 48 (2.1%) showed a relapse at 11 months. All relapsed/refractory patients who achieved a response with checkpoint inhibitors are still in continuous complete response with a median follow-up of 14 months. Data that we have gathered over a 30-year experience in the treatment of primary mediastinal B cell lymphoma patients clearly indicate that a third-generation chemotherapy regimen such as MACOP-B is feasible and easily deliverable on an outpatient basis. Regarding the unmet medical need of relapsed/refractory patients, new encouraging results occurred with the advent of the checkpoint inhibitors.

## Background

Primary mediastinal B cell lymphoma (PMBCL) is a rare subtype of non-Hodgkin lymphoma with unique clinical and molecular characteristics [1]. The 1994 Revised European American Lymphoma Classification firstly recognizes PMLBCL as a subtype of diffuse large B cell lymphoma (DLBCL), although it has been regarded as a specific clinical and biological entity since the 2001 World Health Organization classification [2, 3].

The disease classically occurs in adolescent and young adult women and presents with bulky mediastinal adenopathy. From a molecular standpoint, PMBCL is distinct from DLBCL and shares many biologic similarities with classical Hodgkin lymphoma, including dysregulation of JAK-STAT and NF-κB signaling and overexpression of PD1 ligands [4–7].

Overall, PMBCL shows a better prognosis if compared to other aggressive lymphomas, namely, germinal center B cell and activated B cell subtypes of DLBCL, and a high rate of response to upfront treatment represents the rule. On the contrary, patients whose disease is refractory to standard treatments or who relapse after an initial response are difficult to manage and generally respond poorly to conventional regimens: For this reason, new drugs with innovative mechanisms of action are urgently needed in this context.

What is currently known about the frontline treatment of PMBCL can be extrapolated from several retrospective and a few prospective reports, since no randomized clinical trial has been performed so far. This mainly depends on the rarity of the disease and the consequent inability to design an adequately powered randomized study to test different first-line approaches. It is however consolidated that an anthracycline-containing regimen should be regarded as the first approach [8, 9]. If on one hand CHOP (cyclophosphamide, doxorubicin, vincristine, and prednisone) regimen has been the mainstay of treatment at several American institutions since the pre-rituximab (R) era, European centers have provided the evidence that a combination of methotrexate, doxorubicin, cyclophosphamide, vincristine, bleomycin, and prednisone (MACOP-B) or alternatively a similar regimen, with etoposide instead of methotrexate (VACOP-B), both administered on a weekly basis, could be superior in efficacy to CHOP [10]. The bulk of these data was mainly produced at Italian institutions and within Italian multi-center collaborations since the beginning of the 1990s [11–16]. Differences between CHOP and “third-generation” regimens have become less pronounced with the advent of R; in fact, chemo-immunotherapy has improved survival outcomes if compared to chemotherapy alone [9].

On the other hand, R added to MACOP-B and VACOP-B has shown a less clear benefit [17, 18]. R added to CHOP in a series of 76 patients has demonstrated better event free survival (EFS) and overall survival (OS) rates if compared to historical controls who received CHOP (80% and 89% versus 47% and 69% at 5 years, respectively), a lower rate of early treatment failure (9% versus 30%), as well as higher complete response (CR) rates and significant reduction of disease progression irrespectively of consolidation with radiotherapy (RT) [19].

Few years ago, in a phase 2 prospective trial involving 51 patients, Dunleavy and colleagues have demonstrated that the R-DA-EPOCH (dose-adjusted etoposide, prednisone, vincristine, cyclophosphamide, doxorubicin) yielded a 5-year EFS and OS of 93% and 97%, respectively [20]. These results appeared better than DA-EPOCH alone, suggesting that the addition of R had significantly improved the outcomes of chemotherapy in this context of patients [21]. Following the publication of these encouraging results, the use of R-DA-EPOCH has significantly increased at many American institutions.

Giulino-Roth and colleagues have reported the outcomes of a large series of patients affected by PMBCL, which included 118 adults (age ≥ 21 years) and 38 children, treated with R-DA-EPOCH. EFS at 3 years was 85.9% for the entire cohort of patients and 87.4% for adults; the OS at 3 years was 95.4% [22].

Particularly, regarding the choice of different chemotherapy regimens to include in the frontline treatment of PMBCL, R-DA-EPOCH is effective in the frontline treatment, sometimes allowing to spare RT, especially if patients display a positron emission tomography (PET)-negative response. Survival functions may appear better than what is observed with R-CHOP (and perhaps R-MACOP-B/R-VACOP-B), although the follow-up is still short and no randomized comparisons have been performed.

In the last few years, two issues have emerged regarding the treatment of PMBCL: (1) the role of RT as an adjuvant strategy for consolidating response to chemotherapy and producing an eradication of the disease. There is a trend towards the reduction of RT use in the last 10 years [23, 24], probably as a result of increased concerns about the risk of RT-induced cardiopulmonary side effects and secondary neoplasm (in particular breast cancer because the most part of PMBCL patients are females with an age less than 40); 2) the exploitation of peculiar pathogenetic mechanisms in the treatment of relapsed and progressive disease (PD), with the purpose of overcoming chemoresistance.

In the present study, we report our 30-year monocentric experience in the first-line treatment of PMBCL patients with 151 consecutive cases to analyze response and outcomes.

## Methods

To perform this population-based retrospective study, our clinical database was searched to find all the consecutive patients with a diagnosis of PMBCL, homogeneously treated with a third-generation MACOP-B chemotherapy regimen. Patients treated with chemotherapy regimens other than MACOP-B were excluded. The study was approved by our institutional board and by our Ethical Committee and has been performed in accordance with the ethical standards as laid down in the 1964 Declaration of Helsinki and its later amendments. Patients were consecutively enrolled to avoid selection bias, and all patients provided written informed consent to collect retrospectively their data. We obtained a special permission (for scientific purpose) from our Ethical Committee to collect even the data of patients who were deceased or lost to follow-up.

## Diagnostic and staging procedures

Diagnostic material was obtained by supraclavicular or transthoracic lymph node mediastinoscopy. All diagnostic material was reviewed, and gray zone lymphomas were excluded. Initial clinical evaluation included physical examination; hematologic and biochemical survey; chest X-ray; computed tomography (CT) scan of the neck, chest, abdomen, and pelvis; and unilateral bone marrow biopsy. PET scan was also performed at baseline in all patients treated after 2001.

The extent of mediastinal disease was defined as mediastinal mass ratio (MMR), which was calculated by measuring the maximum single horizontal width of the mass on a standing chest radiograph and dividing it by the maximum intrathoracic diameter. An MMR exceeding one-third or a mass measuring more than 10 cm in its largest diameter as measured by the CT scan was considered bulky.

## Disease restaging and response assessment

Radiologic restaging was performed by total body CT scan 1 month after the end of immuno-/chemotherapy, and then 3 months after the completion of RT as applicable. PET scan was done at the same timepoints, whenever available. Bone marrow biopsy was repeated only if positive for lymphoma at baseline. Treatment responses were categorized according to standardized response criteria [15, 25, 26]. Nodal residues larger than 1.5 cm which have regressed by more than 75% in their major diameter were compatible with a complete response (CR), and regarded as residual scar tissue. PET negativity was corroborative of a CR.

## Statistical analysis

No formal sample size estimation and power calculation were made for this observational retrospective study as we consecutively enrolled all PMBCL patients who underwent MACOP-B as a first-line approach. Part of the cohort was the same used in our previous report which had the aim to assess the difference in the first-line approach (i.e., chemotherapy, radiotherapy, rituximab, or their combinations) [27]. The objective response rate (ORR) was calculated as the sum of partial and complete response rates. OS was calculated from diagnosis to the last follow-up or death for any cause; progression-free survival (PFS) was calculated from start of MACOP-B to the first disease progression or death; disease-free survival (DFS) was determined in all CR patients as the time between the first documented response and the first disease relapse, or death as a result of lymphoma or acute treatment toxicity [26]. Time from first progression to death was also estimated for relapsed/refractory patients. Survival analysis was conducted according to Kaplan-Meier’s method, and log rank test was used for comparisons [28]. Demographics and patients’ characteristics were summarized by descriptive statistics. Statistical analyses were performed with Stata11 (StataCorp LP, TX).

## Results

### Patients’ characteristics and disposition

Between October 1989 and April 2018, 151 patients with de novo PMLBCL were diagnosed and subsequently treated in our institution. The median age at presentation was 34.4 (range 15.7–81.6) years; 91 patients were females and 60 males. Four patients (2.7%) presented with stage I disease, 94 (62.3%) with stage II, 11 (7.3%) with stage III, and 42 (27.8%) with stage IV disease, with lung, spleen, and kidney involvement. B symptoms were present in 63 (41.7%) patients; bulky disease was detected in 137 (90.7%) patients, with a superior vena cava syndrome in 58 (38.4%) patients (Table 1).

**Table 1:** Patients’ characteristics

	Total population
Patients, *n*	151
Males, *n* (%)	60 (39.7)
Females, *n* (%)	91 (60.3)
Median age at diagnosis, years (range)	34.4 (15.7–81.6)
Stage at diagnosis, *n* (%)	
I	4 (2.6)
II	94 (62.3)
III	11 (7.3)
IV	42 (27.8)
B-symptoms, *n* (%)	63 (41.7)
Bulky disease, *n* (%)	137 (90.7)
Superior vena cava syndrome, *n* (%)	58 (38.4)

All patients were treated with the MACOP-B regimen, given for 12 consecutive weeks, with leucovorin rescue after any methotrexate-containing cycle [10]. The median number of cycles delivered was 12. R was administered every 21 days (375 mg/m^2^) along with chemotherapy in 57 patients (58.2%), all treated after 2001, when it became available in Italy. Globally, 111/151 (73.5%) received R (patients treated from 2001). Eighty-seven (57.6%) patients received mediastinal radiotherapy (RT), 4 to 6 weeks after the completion of (immuno)chemotherapy, with doses ranging from 30 to 36 Gy over a 4- to 5-week treatment schedule, with fractions of 180 cGy/day for 5 days per week. The decision to use RT was based on era-specific institutional guidelines: it was routinely administered after chemotherapy in all patients since 1993 to 2002; before 1993, it was delivered upon physician’s discretion; after 2002, along with the use of PET in detecting potential residual masses after chemotherapy, RT was not administered in those patients with a negative PET scan (i.e., total absence of disease, also minimal residual disease).

### Induction treatment

**Fig. 1:**
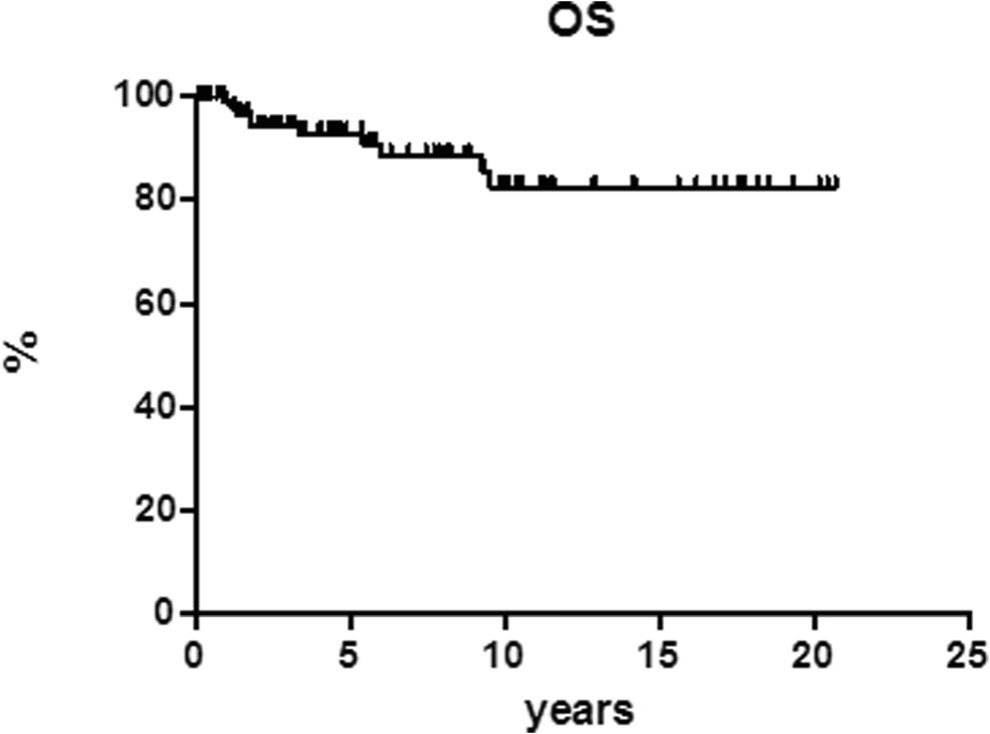
Overall survival (OS)

**Fig. 2:**
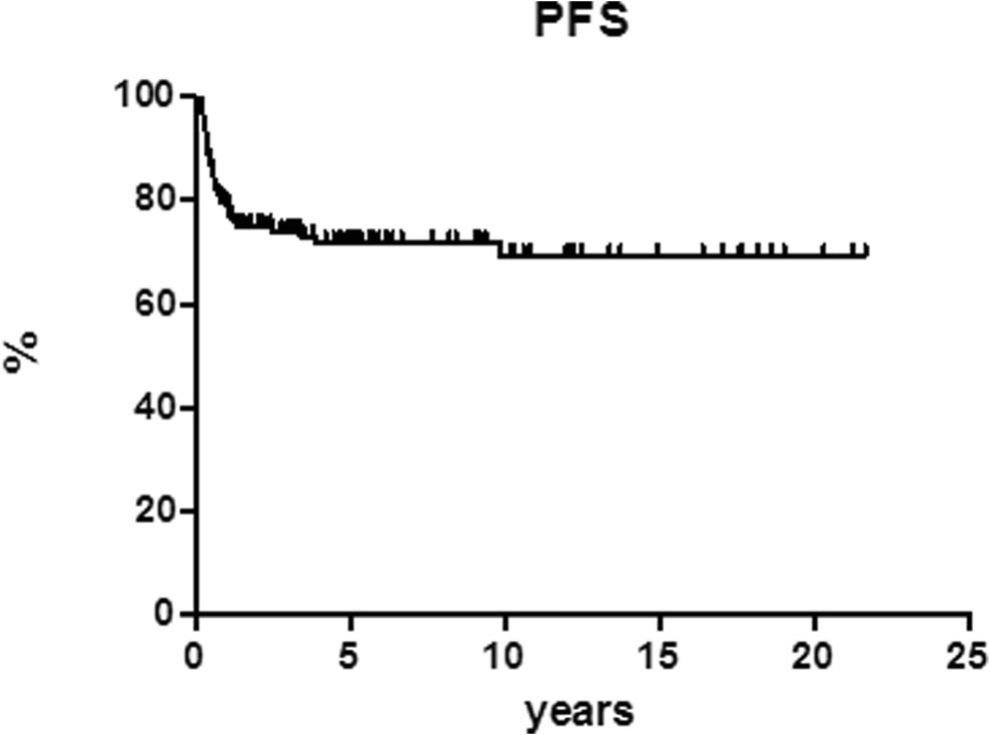
Progression-free survival (PFS)

**Fig. 3:**
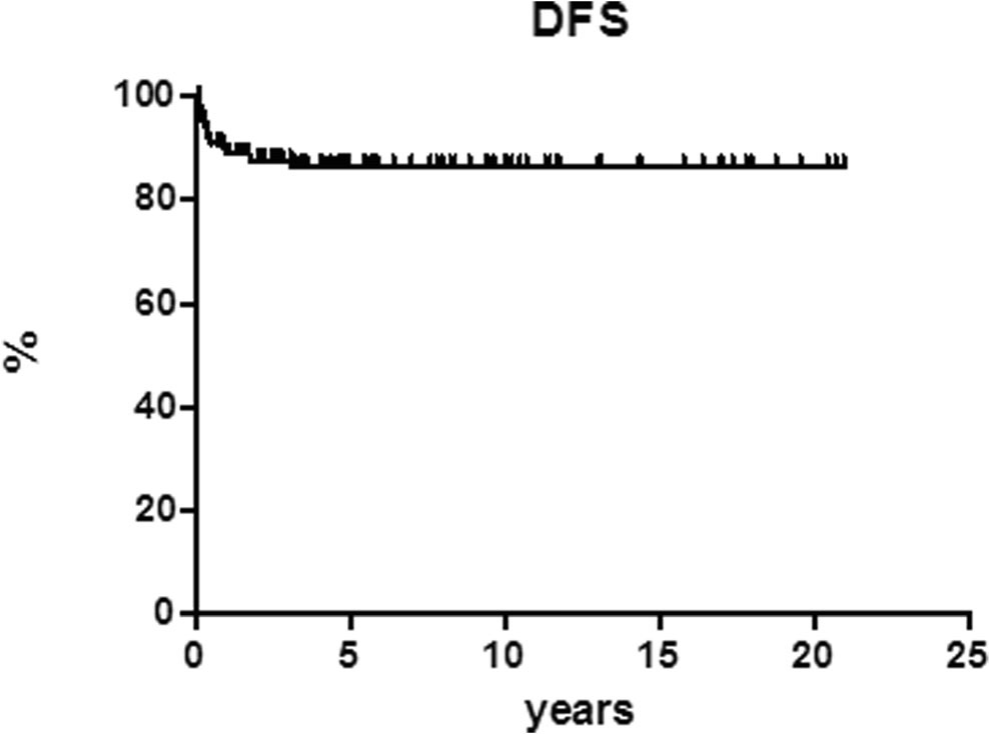
Disease-free survival (DFS)

After 12 cycles of MACOP-B regimen (with or without R), 120 patients out of 151 (79.5%) achieved a CR and 12 (7.9%) a partial response (PR) leading to an ORR of 87.4%; a stable disease (SD) was documented in two patients and 17 PD. Median follow-up duration for the entire cohort of patients is 5.5 years (range, 1.5–21 years). Among patients who obtained a CR, 107 out of 120 (89.2%) are in continuous CR (CCR). Only 13 of the 120 CRs (10.8%) showed disease relapse with a median duration of response of 3.9 months (range, 2–36 months); in particular, all but one (that relapsed at 3 years) showed a relapse within the first 15 months. The projected OS at 21 years for all the patients is 82.6% (Fig. 1), with a PFS of 69.3% (Fig. 2) and a DFS of 86.4% (Fig. 3). All curves show a plateau. Survivals and patients at risk at 5, 10, and 15 years are reported in Table 2.

**Table 2:** Survival proportions and patients at risk

	Overall survival		Disease-free survival		Progression-free survival	
	%	*n* at risk	%	*n* at risk	%	*n* at risk
5 years	92.9	49	86.4	45	71.8	54
10 years	82.6	26	86.4	26	69.3	28
15 years	82.6	14	86.4	14	69.3	14

No difference for CR rates and survivals was observed basing on the use of rituximab or patient stage.

We observed two central nervous system relapses (with concomitant nodal disease presentation) 2 and 3 months after end of first-line therapy, respectively.

### The role of radiotherapy

Since 2002 along with the use of PET in detecting potential residual masses after chemotherapy, we have decided to not administer RT in those patients presenting a negative PET scan after MACOP-B. The percentage of PET-negative patients undergoing RT has gone to zero over time. Globally, only one out of the 48 CR patients who underwent RT (2.1%) presented a disease relapse (at 11 months). Four patients with a CR status for lymphoma deceased due to a second malignancy.

### Relapsed/refractory patients

Considering patients who obtained PR after the induction treatment, CR patients who relapsed, and the refractory patients (including patients in SD or PD at the end of the frontline therapy), the total amount of these 3 cohorts was represented by 44 patients. Five of these 44 patients died rapidly (within 2 months) due to PD. Among the remaining 39 patients, the salvage treatment decision was based on era-specific institutional guidelines related to the therapeutic approaches available.

**Fig. 4:**
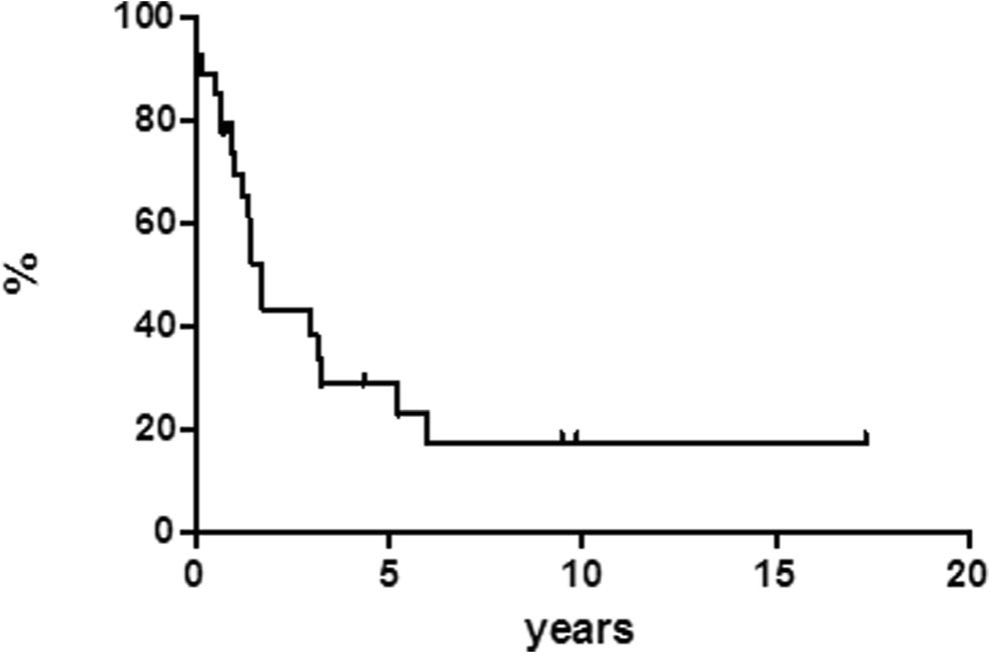
Time from first progression to death (relapsed patients)

In fact, we had two subsets of patients: (1) patients who received salvage conventional immune-chemotherapy regimens with the potential consolidation with autotransplant and/or allotransplant (12 patients) and (2) patients who received the same specific treatment modality like the first cohort and, in addition, pembrolizumab or nivolumab plus brentuximab vedotin (27 patients). In the first cohort, only 2 out of 12 patients (16.7%) obtained a response—both PR—and only one of them converted from PR to CR after consolidation with autologous stem cell transplantation, still in CCR after 45 months. In the second cohort, 27 patients received checkpoint inhibitors after conventional salvage treatments. All these patients were refractory to the last treatment with a median number of previous therapies of 3 (range 2–8). Eighteen patients received pembrolizumab and 9 nivolumab plus brentuximab vedotin. Among patients who received pembrolizumab, 6 out of 18 (30%) obtained a CR; with a median follow-up of 18 months (range 28–44), all these 6 patients are still in CCR (none of them received allotransplant consolidation). Regarding the patients who received nivolumab plus brentuximab vedotin, 4 out of 9 (44.4%) showed a CR, and, in particular, all of them are in CCR with a median follow-up of 10 months (range 12–29). Median time from first progression to death for all relapsed/refractory patients was reached at 1.7 years (Fig. 4).

## Discussion

The peculiar characteristics of PMBCL, the high chances of cure documented in the last 30 years by international experiences, and the long disease-free life expectancy of cured patients have always drawn attention in finding out the most feasible frontline treatment and the most convenient combination of treatment modalities, with the intent to maximize the long-term clinical outcomes and, at the same time, to decrease the potential late consequences of highly toxic combined treatments [29, 30].

Our monocentric experience over a period of more than 30 years ideally encompasses all the issues met by treating physicians throughout the years and may virtually suggest some still open points which require clarification with further ad hoc studies. We reported on a series of 151 patients homogeneously treated with a weekly “third-generation” schedule. Part of the cohort also received external mediastinal RT and/or anti-CD20 immunotherapy, depending on our institutional era-specific guidelines. The addition of rituximab did not imply a clear advantage as previously reported [27].

In our experience, the MACOP-B (with/without R) regimen yielded an ORR of 87.4% with a CR rate of 79.5%. Among patients who obtained a CR, 107 out of 120 (89.2%) are in CCR at the latest available follow-up. Thirteen patients showed disease relapse after a median duration of response of 3.9 months (range, 2–36 months). The projected OS at 21 years for all the patients is 82.6%, with a PFS of 69.3% and a DFS of 86.4%, and all these curves showed a plateau.

Regarding the indirect comparison with R-DA-EPOCH regimen data, the 79.5% of CR rate of our series is a good result if compared with the CR rates reported in literature: 96%, 75%, and 84%, respectively [20, 22, 31]. In addition, our DFS curve shows a plateau at 86.4% and represents a very good comparator for the historical DA-EPOCH-R curves: 93% EFS at 5 years, 86% EFS at 3 years, and 85% PFS at 2 years, respectively.

Although there is evidence of RT efficacy in those with residual disease following R-CHOP or V/MACOP-B, the need for RT in patients who have a CR is uncertain. PET scan may offer the opportunity to identify patients in which RT can be spared.

A negative end of treatment (EOT) PET has been successfully used to detect patients who can safely forego RT following R-CHOP and R-MACOP-B [23, 24]. Both retrospective studies demonstrated no difference in outcome for PET-negative patients who did not receive RT compared to PET-positive patients that did, suggesting that RT is not needed for PET-negative patients. Fortunately, a randomized trial is in progress to conclusively answer to this question (IELSG-37): Patients who have a negative EOT-PET following R-containing immunochemotherapy are randomized to either mediastinal radiation or close observation [32]. This trial should be able to demonstrate a non-inferior outcome in patients not receiving RT; the study may eventually allow to individualize treatment for each patient by adapting it to the response documented by PET. This may allow limiting the indication for RT only to the PET-positive patients who have an inadequate response to chemo-immunotherapy. With our report, we confirmed and strengthened data previously published although with the well-known bias of retrospective studies [24]; only one out of 48 PET-negative patients (2.1%) at the end of the chemo-immunotherapy presented a disease relapse (at 11 months).

The unique biological features of PMBCL are providing clues to designing new treatment strategies for relapsed and refractory disease, which is characterized by a dismal prognosis and still represents a truly unmet medical need. For patients with primary refractory or relapsed disease, outcomes are poor [33].

There have been many advances in understanding the molecular biology of this lymphoma, and, in particular, the discovery of the role of PD1 ligands and JAK-STAT pathways has paved the way for the investigation of novel therapies and approaches in PMBCL. In fact, pembrolizumab as a single agent and nivolumab in combination with brentuximab vedotin have showed very interesting results in heavily pretreated PMBCL patients [34–36]. In this present report, we included patients treated in the abovementioned studies, and 10 out of 27 patients (37%) obtained a CR and were all in CCR without any kind of consolidation approach at the latest available follow-up.

In conclusion, data that we have gathered over a 30-year experience in the first-line treatment of PMBCL patients clearly indicate that a “third-generation” chemotherapy regimen such as MACOP-B is feasible and easily deliverable on an outpatient basis. Its efficacy in terms of CR rate and DFS is quite similar to the R-DA-EPOCH published data. Radiotherapy in this context remains a powerful strategy to convert PRs to CRs, but it may be spared in patients obtaining a PET-documented CR after chemo-immunotherapy without any harmful prognostic consequences. Regarding the unmet medical need of relapsed/refractory patients, the tunnel is no longer so dark with the advent of the checkpoint inhibitors.

## Data Availability

The datasets used and analyzed during the current study are available from the corresponding authors on reasonable request.

## References

[1] Savage KJ, Monti S, Kutok JL, Cattoretti G, Neuberg D, de Leval L, Kurtin P, Dal Cin P, Ladd C, Feuerhake F, Aguiar RC, Li S, Salles G, Berger F, Jing W, Pinkus GS, Habermann T, Dalla-Favera R, Harris NL, Aster JC, Golub TR, Shipp MA (2003). The molecular signature of mediastinal large B-cell lymphoma differs from that of other diffuse large B-cell lymphomas and shares features with classical Hodgkin lymphoma. Blood.

[2] Harris NL, Jaffe ES, Stein H, Banks PM, Chan JK, Cleary ML, Delsol G, de Wolf- Peeters C, Falini B, Gatter KC (1994). A revised European-American classification of lymphoid neoplasms: a proposal from the international lymphoma study group. Blood.

[3] Banks PM, Warnke RA, Jaffe ES, Harris NL, Stein H, Wardiman JW (2001). Mediastinal (thymic) large B-cell lymphoma. World Health Organization classification of tumors. Pathology and genetics of tumors of hematopoietic and lymphoid tissues.

[4] Feuerhake F, Kutok JL, Monti S, Chen W, LaCasce A, Cattoretti G, Kurtin P, Pinkus GS, de Leval L, Harris NL, Savage KJ, Neuberg D, Habermann TM, Dalla-Favera R, Golub TR, Aster JC, Shipp MA (2005). NFkappaB activity, function, and target-gene signatures in primary mediastinal large B-cell lymphoma and diffuse large B-cell lymphoma subtypes. Blood.

[5] Guiter C, Dusanter-Fourt I, Copie-Bergman C, Boulland ML, le Gouvello S, Gaulard P, Leroy K, Castellano F (2004). Constitutive STAT6 activation in primary mediastinal large B-cell lymphoma. Blood.

[6] Moller P, Lammler B, Herrmann B, Otto HF, Moldenhauer G, Momburg F (1986). The primary mediastinal clear cell lymphoma of B-cell type has variable defects in MHC antigen expression. Immunology.

[7] Rosenwald A, Wright G, Leroy K, Yu X, Gaulard P, Gascoyne RD, Chan WC, Zhao T, Haioun C, Greiner TC, Weisenburger DD, Lynch JC, Vose J, Armitage JO, Smeland EB, Kvaloy S, Holte H, Delabie J, Campo E, Montserrat E, Lopez-Guillermo A, Ott G, Muller-Hermelink HK, Connors JM, Braziel R, Grogan TM, Fisher RI, Miller TP, LeBlanc M, Chiorazzi M, Zhao H, Yang L, Powell J, Wilson WH, Jaffe ES, Simon R, Klausner RD, Staudt LM (2003). Molecular diagnosis of primary mediastinal B cell lymphoma identifies a clinically favorable subgroup of diffuse large B cell lymphoma related to Hodgkin lymphoma. J Exp Med.

[8] Zinzani PL, Martelli M, Poletti V, Vitolo U, Gobbi PG, Chisesi T, Barosi G, Ferreri AJ, Marchetti M, Pimpinelli N, Tura S, Italian Society of Hematology, Italian Society of Experimental Hematology, Italian Group for Bone Marrow Transplantation (2008). Practice guidelines for the management of extranodal non-Hodgkin’s lymphomas of adult non-immunodeficient patients. Part I: primary lung and mediastinal lymphomas. A project of the Italian Society of Hematology, the Italian society of experimental hematology and the Italian Group for Bone Marrow Transplantation. Haematologica.

[9] Savage KJ, Al-Rajhi N, Voss N (2006). Favorable outcome of primary mediastinal large B-cell lymphoma in a single institution: the British Columbia experience. Ann Oncol.

[10] Klimo P, Connors JM (1985). MACOP-B chemotherapy for the treatment of diffuse large B-cell lymphoma. Ann Intern Med.

[11] Zinzani PL, Bendandi M, Frezza G, Gherlinzoni F, Merla E, Salvucci M, Magagnoli M, Babini L, Tura S (1996). Primary mediastinal B-cell lymphoma with sclerosis: clinical and therapeutic evaluation of 22 patients. Leuk Lymphoma.

[12] Lazzarino M, Orlandi E, Paulli M, Boveri E, Morra E, Brusamolino E, Kindl S, Rosso R, Astori C, Buonanno MC (1993). Primary mediastinal B-cell lymphoma with sclerosis: an aggressive tumor with distinctive clinical and pathologic features. J Clin Oncol.

[13] Lazzarino M, Orlandi E, Paulli M, Sträter J, Klersy C, Gianelli U, Gargantini L, Rousset MT, Gambacorta M, Marra E, Lavabre-Bertrand T, Magrini U, Manegold C, Bernasconi C, Möller P (1997). Treatment outcome and prognostic factors for primary mediastinal (thymic) B-cell lymphoma: a multicenter study of 106 patients. J Clin Oncol.

[14] Todeschini G, Secchi S, Morra E, Vitolo U, Orlandi E, Pasini F, Gallo E, Ambrosetti A, Tecchio C, Tarella C, Gabbas A, Gallamini A, Gargantini L, Pizzuti M, Fioritoni G, Gottin L, Rossi G, Lazzarino M, Menestrina F, Paulli M, Palestro M, Cabras MG, di Vito F, Pizzolo G (2004). Primary mediastinal large B-cell lymphoma (PMLBCL): long term results from a retrospective multicenter Italian experience in 138 patients treated with CHOP or MACOP-B/VACOP-B. Br J Cancer.

[15] Zinzani PL, Martelli M, Magagnoli M, Pescarmona E, Scaramucci L, Palombi F, Bendandi M, Martelli MP, Ascani S, Orcioni GF, Pileri SA, Mandelli F, Tura S (1999). Treatment and clinical management of primary mediastinal large B-cell lymphoma with sclerosis: MACOP-B regimen and mediastinal radiotherapy monitored by (67) gallium scan in 50 patients. Blood.

[16] Zinzani PL, Martelli M, Bendandi M, de Renzo A, Zaccaria A, Pavone E, Bocchia M, Falini B, Gobbi M, Gherlinzoni F, Stefoni V, Tani M, Tura S (2001). Primary mediastinal large B-cell lymphoma with sclerosis: a clinical study of 89 patients treated with MACOP-B chemotherapy and radiation therapy. Haematologica.

[17] Zinzani PL, Stefoni V, Finolezzi E, Brusamolino E, Cabras MG, Chiappella A, Salvi F, Rossi A, Broccoli A, Martelli M (2009). Rituximab combined with MACOP-B or VACOP-B and radiation therapy in primary mediastinal large B-cell lymphoma: a retrospective study. Clin Lymphoma Myeloma.

[18] Avigdor A, Sirotkin T, Kedmi M, Ribakovsy E, Berkowicz M, Davidovitz Y, Kneller A, Merkel D, Volchek Y, Davidson T, Goshen E, Apter S, Shimoni A, Ben-Bassat I, Nagler A (2014). The impact of R-VACOP-B and interim FDG-PET/CT on outcome in primary mediastinal large B cell lymphoma. Ann Hematol.

[19] Vassilakopoulos TP, Pangalis GA, Katsigiannis A (2012). Rituximab, cyclophosphamide, doxorubicin, vincristine, and prednisone with or without radiotherapy in primary mediastinal large B-cell lymphoma: the emerging standard of care. Oncol.

[20] Dunleavy K, Pittaluga S, Maeda LS, Advani R, Chen CC, Hessler J, Steinberg SM, Grant C, Wright G, Varma G, Staudt LM, Jaffe ES, Wilson WH (2013). Dose-adjusted EPOCH-rituximab therapy in primary mediastinal B-cell lymphoma. N Engl J Med.

[21] Wilson WH, Grossbard ML, Pittaluga S, Cole D, Pearson D, Drbohlav N, Steinberg SM, Little RF, Janik J, Gutierrez M, Raffeld M, Staudt L, Cheson BD, Longo DL, Harris N, Jaffe ES, Chabner BA, Wittes R, Balis F (2002). Dose-adjusted EPOCH chemotherapy for untreated large B-cell lymphomas: a pharmacodynamic approach with high efficacy. Blood.

[22] Giulino-Roth L, O'Donohue T, Chen Z, Bartlett NL, LaCasce A, Martin-Doyle W, Barth MJ, Davies K, Blum KA, Christian B, Casulo C, Smith SM, Godfrey J, Termuhlen A, Oberley MJ, Alexander S, Weitzman S, Appel B, Mizukawa B, Svoboda J, Afify Z, Pauly M, Dave H, Gardner R, Stephens DM, Zeitler WA, Forlenza C, Levine J, Williams ME, Sima JL, Bollard CM, Leonard JP (2017). Outcomes of adults and children with primary mediastinal B-cell lymphoma treated with dose-adjusted EPOCH-R. Br J Haematol.

[23] Savage K, Yenson P, Shenkier T (2012). The outcome of primary mediastinal large B-cell lymphoma (PMBCL) in the R-CHOP treatment era. Blood.

[24] Zinzani PL, Broccoli A, Casadei B, Stefoni V, Pellegrini C, Gandolfi L, Maglie R, Argnani L, Pileri S, Fanti S (2015). The role of rituximab and positron emission tomography in the treatment of primary mediastinal lymphoma: experience on 74 patients. Hematol Oncol.

[25] Cheson BD, Horning SJ, Coiffier B (1999). Report of an international workshop to standardized response criteria for non-Hodgkin's lymphomas. NCI Sponsored International Working Group. J Clin Oncol.

[26] Cheson BD, Pfistner B, Juweid ME, Gascoyne RD, Specht L, Horning SJ, Coiffier B, Fisher RI, Hagenbeek A, Zucca E, Rosen ST, Stroobants S, Lister TA, Hoppe RT, Dreyling M, Tobinai K, Vose JM, Connors JM, Federico M, Diehl V, International Harmonization Project on Lymphoma (2007). Revised response criteria for malignant lymphoma. J Clin Oncol.

[27] Broccoli A, Casadei B, Stefoni V, Pellegrini C, Quirini F, Tonialini L, Morigi A, Marangon M, Argnani L, Zinzani PL (2017). The treatment of primary mediastinal large B-cell lymphoma: a two decades monocentric experience with 98 patients. BMC Cancer.

[28] Kaplan EL, Meier P (1958). Non-parametric estimation from incomplete observations. J Am Stat Assoc.

[29] Zinzani PL, Piccaluga PP (2011). Primary mediastinal DLBCL: evolving biologic understanding and therapeutic strategies. Curr Oncol Rep.

[30] Zinzani PL, Broccoli A (2016). Optimizing outcomes in primary mediastinal B-cell lymphoma. Hematol Oncol Clin North Am.

[31] Shah NN, Szabo A, Huntington SF, Epperla N, Reddy N, Ganguly S, Vose J, Obiozor C, Faruqi F, Kovach AE, Costa LJ, Xavier AC, Okal R, Kanate AS, Ghosh N, Kharfan-Dabaja MA, Strelec L, Hamadani M, Fenske TS, Calzada O, Cohen JB, Chavez J, Svoboda J (2018). R-CHOP versus dose-adjusted R-EPOCH in frontline management of primary mediastinal B-cell lymphoma: a multi-centre analysis. Br J Haematol.

[32] Martelli M, Zucca E, Gospodarowicz M (2013). A randomized multicenter, two arm phase III comparative study assessing the role of mediastinal radiotherapy after rituximab-containing chemotherapy regimens to patients with newly diagnosed primary mediastinal large B-cell lymphoma (PMBCL): the IELSG 37 study. Hematol Oncol.

[33] Kuruvilla J, Pintilie M, Tsang R, Nagy T, Keating A, Crump M (2008). Salvage chemotherapy and autologous stem cell transplantation are inferior for relapsed or refractory primary mediastinal large B-cell lymphoma compared with diffuse large B-cell lymphoma. Leuk Lymphoma.

[34] Zinzani PL, Ribrag V, Moskowitz CH, Michot JM, Kuruvilla J, Balakumaran A, Zhang Y, Chlosta S, Shipp MA, Armand P (2017). Safety and tolerability of pembrolizumab in patients with relapsed/refractory primary mediastinal large B-cell lymphoma. Blood.

[35] Zinzani PL, Santoro A, Gritti G, Brice P, Barr PM, Kuruvilla J, Cunningham D, Kline J, Johnson NA, Mehta-Shah N, Manley T, Francis S, Sharma M, Moskowitz AJ (2019). Nivolumab combined with brentuximab vedotin for relapsed/refractory primary Mediastinal large B-cell lymphoma: efficacy and safety from the phase II CheckMate 436 study. J Clin Oncol.

[36] Armand P, Rodig S, Melnichenko V, Thieblemont C, Bouabdallah K, Tumyan G, Özcan M, Portino S, Fogliatto L, Caballero MD, Walewski J, Gulbas Z, Ribrag V, Christian B, Perini GF, Salles G, Svoboda J, Zain J, Patel S, Chen PH, Ligon AH, Ouyang J, Neuberg D, Redd R, Chatterjee A, Balakumaran A, Orlowski R, Shipp M, Zinzani PL (2019). Pembrolizumab in relapsed or refractory primary mediastinal large B-cell lymphoma. J Clin Oncol.

